# Learning New Vocabulary Implicitly During Sleep Transfers With Cross-Modal Generalization Into Wakefulness

**DOI:** 10.3389/fnins.2022.801666

**Published:** 2022-03-09

**Authors:** Matthieu Koroma, Maxime Elbaz, Damien Léger, Sid Kouider

**Affiliations:** ^1^Brain and Consciousness Group (ENS, EHESS, CNRS), Département d’Études Cognitives, École Normale Supérieure, Paris, France; ^2^École Doctorale Cerveau Cognition Comportement, Université Pierre et Marie Curie Sorbonne Universités, Paris, France; ^3^Université de Paris, APHP, Hôtel-Dieu de Paris, Centre du Sommeil et de la Vigilance, EA 7330 VIFASOM Sommeil-Vigilance-Fatigue et Santé Publique, Paris, France

**Keywords:** associative learning, vocabulary acquisition, implicit memory, cross-modal generalization, sleep, EEG, slow wave, associative transfer

## Abstract

New information can be learned during sleep but the extent to which we can access this knowledge after awakening is far less understood. Using a novel Associative Transfer Learning paradigm, we show that, after hearing unknown Japanese words with sounds referring to their meaning during sleep, participants could identify the images depicting the meaning of newly acquired Japanese words after awakening (*N* = 22). Moreover, we demonstrate that this cross-modal generalization is implicit, meaning that participants remain unaware of this knowledge. Using electroencephalography, we further show that frontal slow-wave responses to auditory stimuli during sleep predicted memory performance after awakening. This neural signature of memory formation gradually emerged over the course of the sleep phase, highlighting the dynamics of associative learning during sleep. This study provides novel evidence that the formation of new associative memories can be traced back to the dynamics of slow-wave responses to stimuli during sleep and that their implicit transfer into wakefulness can be generalized across sensory modalities.

## Introduction

It is well established that memories acquired during the day can be consolidated during sleep (Walker et al., 2003; Rasch and Born, 2013). Whether new information can be acquired during sleep has, however, given rise to controversial or inconsistent results until recently [e.g., Bruce et al., 1970; see Peigneux et al. (2001) for a review]. In the past years, a series of convergent findings have demonstrated that learning during sleep is a reality, although its extent and flexibility remain to be clarified [Arzi et al., 2012; see Puchkova (2020) for a review]. Evidence of learning during sleep was initially limited to fairly simple mechanisms such as sensory conditioning (Arzi et al., 2012, 2014) and perceptual encoding (Ruch et al., 2014; Andrillon and Kouider, 2016; Andrillon et al., 2017). Recent results reveal that verbal associations can also be acquired during sleep, demonstrating that the learning abilities of the sleeping brain can extend to higher levels of representations (Züst et al., 2019).

Yet, a remaining issue concerns the flexibility of learning during sleep. In particular, it is still unclear whether humans can generalize to new domains on the basis of knowledge acquired during sleep and use it in their behavior during wakefulness (Ruch and Henke, 2020). This ability, known as transfer learning, is a core aspect of neural plasticity and is considered a key determinant of flexible learning in both humans and machines (Torrey and Shavlik, 2010; Lake et al., 2017). In this study, we aimed at testing the hypothesis that memory systems during sleep not only support the acquisition of new information but are also able to transfer this information across sensory domains and influence subsequent behavior during wakefulness.

To address this issue, we relied on a novel Associative Transfer Learning (ATL) paradigm. During sleep, participants were presented with words in a foreign tongue (Japanese) concomitantly with the acoustic representation of their meaning (e.g., the Japanese equivalent of the word “dog” along with the sound of a barking dog) while we recorded their electroencephalograph (EEG). Upon awakening, the Japanese words were played again while participants had to select the corresponding image in a two-alternative forced-choice task. This paradigm allowed us to test not only whether the mapping from a new verbal representation and its acoustic meaning could be acquired during sleep, but also whether this mapping could be transferred to the pictorial representation of their meaning during wakefulness.

To elucidate the neural mechanisms involved in ATL, we investigated whether EEG responses during sleep predicted memory performance after awakening. To do so, we compared EEG responses during sleep between items that were correctly identified during the memory test and those that led to incorrect decisions. Züst et al. (2019) previously identified a neural correlate of memory acquisition in the slow-wave activity in response to stimulations. Brain entrainment at a slow-wave frequency was also observed for sensory conditioning and interestingly changed throughout the sleep learning process (Canales-Johnson et al., 2020). Here, we analyzed neural responses to sounds at the beginning and the end of the sleep learning process. We hypothesized that slow-wave activity would predict items that were later remembered, and that this neural signature would increase over night, reflecting the dynamics of sleep learning.

## Materials and Methods

### Participants

Twenty-five French-native speakers (age: 24,1, min: 20, max: 34, 16 females) were recruited for this study based on an online questionnaire ensuring they could easily fall asleep in a noisy environment and had no prior knowledge of Japanese nor related languages (Chinese, Korean). Sample size was chosen based on previous studies investigating learning during sleep (Strauss et al., 2015; Andrillon et al., 2017). Participants were interviewed by a sleep doctor to ensure that they had no history of hearing nor sleep disorders. Participants were monitored 3–10 days before the experiment by actimetry (FitBit Charge HR) and filled a sleep agenda to ensure a normal sleep schedule before the experiment. Additionally, participants were deprived from stimulants (e.g., caffeine) the day of the experiment. Two participants were excluded because of difficulties in falling asleep with auditory stimulations and one due to technical issues, resulting in twenty-two participants (age: 23,7, min: 20, max: 29, 15 females). The present protocol has been approved by the local ethical committee (Comité de Protection des Personnes, Ile-de-France I, Paris, France) and all participants provided informed consent.

### Stimuli

Totally 48 Japanese words were first selected from three categories (animals, natural elements, body parts or bodily actions; see Supplementary Table 1 for the full list of words). Auditory versions of Japanese words were generated using a licensed-free online text-to-speech software^1^. Sounds corresponding to Japanese words were extracted from licensed-free online audio banks^2^,^3^ and matched in length using the Matlab (Mathworks Inc.) implementation of the VSOLA (variable parameter synchronized overlap add) algorithm (duration = 2.58 s) (Dorran et al., 2003). Auditory stimuli were matched in intensity by normalizing their root mean square. Stimuli were delivered to participants through speakers placed at the head of the bed. During sleep, Japanese words were played simultaneously with the sound corresponding to their meaning (e.g., the Japanese equivalent of dog with the barking of a dog). To ensure successful encoding of Japanese words, they were played twice louder than their corresponding sound and repeated twice with half second intervals. This resulted in 48 sound-word pairs matched in length using the VSOLA algorithm (first word onset after sound onset = 0.5 s, second word onset = 1.60 s ± 0.099 (mean ± STD), sound duration = 2.66 s) and in intensity by normalizing their root mean square. A corresponding image for each Japanese word was selected online and their display was matched in size on the monitor. This procedure resulted in 48 associations between a Japanese word (e.g., the Japanese equivalent of dog), a corresponding sound (e.g., barking of a dog) and a corresponding image (e.g., the picture of a dog). For each participant, the 48 associations were then randomly assigned to 3 lists of 16 items (NREM, REM and control lists).

### Experimental Protocol

**FIGURE 1:**
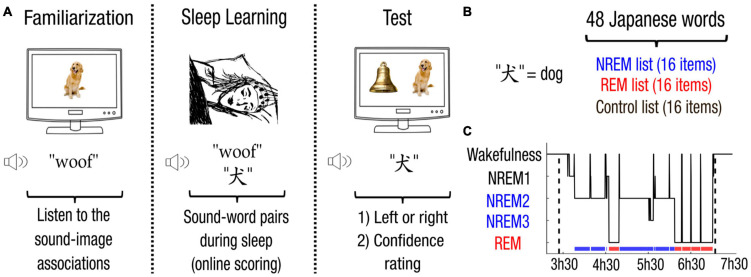
Associative Transfer Learning paradigm. **(A)** During the first phase performed awake, participants were familiarized with the 48 sound-image pairs. Participants were then allowed to fall asleep. Whenever participants entered NREM or REM sleep, sounds from the corresponding list were played simultaneously with their corresponding Japanese translations. Upon awakening, participants performed a memory test. Japanese words were played with two images belonging to the same list (i.e., NREM, REM, or control list). Participants first chose which image correctly depicts the Japanese word (left or right), and then provided their confidence on a three-level scale (null, low, high). **(B)** For each participant, the 48 Japanese words (e.g., the Japanese equivalent for “dog”) were randomly assigned into 3 lists, resulting in a NREM, a REM and a control list of 16 items each. **(C)** Sleep scoring (hypnogram) of the experiment for one participant.

Participants were invited to the sleep lab (Centre du Sommeil et de la Vigilance, Hotel-Dieu, Paris) the evening prior to the experiment. Participants were then equipped with a 64-channel electroencephalography (EEG) gel-net (EGI system, Electrical Geodesic Inc.) and chin electromyography (EMG). The first part of the night was devoted to a different task orthogonal to the purpose of the present experiment. Participants were awakened at 3 am to start the experiment (Figure 1A). During the familiarization phase, 48 sound-image pairs (e.g., a picture of a dog and the barking sound “*woof*”) were played four times in a randomized order. Participants were instructed to remember the sound-image associations. During the sleep learning phase, participants were then allowed to fall asleep with the volume set around 50 dB and adjusted according to participant’s preference following previous experiments (Strauss et al., 2015; Andrillon et al., 2017). While participants were scored as awake or in transition to sleep (i.e., still in NREM1), sounds alone (e.g., “*woof*”) were continuously played separated by an interstimulus interval of 4–6 s uniformly distributed random jitter. Whenever participants entered NREM or REM sleep, Japanese translations were added on top of their corresponding sounds (e.g., the Japanese word for dog with the barking sound “*woof*”). Stimuli from different lists were played during NREM and REM sleep (Figures 1B,C and Supplementary Table 2 for stimulation statistics across participants). Participants woke up at 7 am and started the test phase whenever they were ready. During the test phase, participants performed a two-Alternative Forced Choice (2AFC) task. Each item was paired with another item from the same list (i.e., either from the NREM, REM or control list). Japanese translations were played twice to ensure successful encoding and facilitate recognition. Two images were displayed among which only one depicted the Japanese word. Participants first decided which image represented the Japanese word, and second assessed their confidence in their response (null if it was a blind guess, low, high). The side (left or right) of the correct image was counterbalanced within lists. The order of apparition of items across all lists was randomly shuffled. Subjects could correct their decision before giving their confidence response to reduce mistakes resulting from wrong button presses during the first decision.

### Electroencephalograph Recordings

Electroencephalograph signals were amplified (NetAmp 300), referenced online to Cz and sampled at 500 Hz. Electrooculograms (EOG) and chin electromyograms (EMG) were recorded with electrodes placed around the eyes and on the chin respectively. For offline analysis, defective channels were first identified and interpolated using neighboring electrodes. EEG and EOG signals were re-referenced to mastoids and bandpass filtered between 0.1 and 30 Hz (two-pass Butterworth filter, 5^th^ order). EMG signals were obtained with a local derivation and band-passed between 80 and 160 Hz (two-pass Butterworth filter, 5^th^ order). EEG recordings were synchronized with audio stimuli using a signal recorded by the amplifier at the onset and offset of each stimulus.

### Sleep Scoring

Stimulus presentation during sleep was adapted online by a trained scorer (M.K.) following established guidelines (Iber et al., 2007). Sleep stages were scored offline on 20-s long windows by trained scorers (M.K. and D.L.) blind to experimental conditions (see Figure 1C for an hypnogram of one participant, Supplementary Table 3 for sleep statistics across participants). Micro-arousals were carefully noted.

### Electroencephalograph Analysis

Electroencephalograph recordings were resampled at 100 Hz for data analysis. EEG responses from −0.5 to 4.5 s around stimulus onset of each trial were selected. Brain potentials were baseline corrected [(−0.2, 0)s]. The mean and standard deviations (SD) of the maximal differences across trials for each electrode was computed. Trials with at least one electrode deviating for more than 3 SD above the mean were rejected to avoid artifactual contamination [5.0%, CI = (4.1, 6.1)]. This resulted in conserving 1,689 trials [CI = (1,619, 1,758)]. Among these trials, only trials that were scored both online and offline were kept for further analysis, resulting in 625 trials in NREM sleep [CI = (540, 711)] per participant (Supplementary Table 2 for the online scoring efficiency). Brain potentials for channels over a frontal cluster of electrodes (Fz, F1, F2, F3, F4, FPz, FP1, FP2, FP3, FP4) were averaged across trials of the same conditions following on a previous study (Züst et al., 2019). To analyze the time-course of brain responses, we relied on non-parametric cluster permutation statistics to control for multiple comparisons. Clusters were defined as consecutive time-points for which parametric tests reached a specific threshold (α = 0.05). For each cluster, the sum of t-values was compared to the maximum cluster statistics obtained after random permutation of the conditions considered (*N* = 1,000 permutations). We computed a Monte-Carlo *P*-value (referred as P_cluster_) and the sum of t-values of clusters with P_cluster_ < 0.05 were reported.

## Results

### Learning During NREM Sleep

**FIGURE 2:**
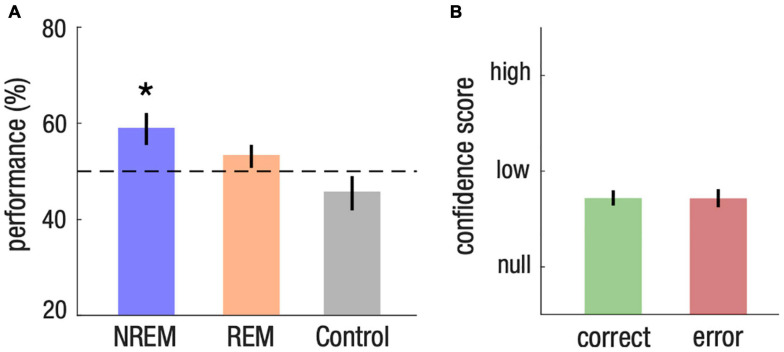
Implicit associative transfer learning for words presented during NREM sleep. **(A)** Memory performance across NREM, REM and control list. Student’s T-test against chance level (50%, dotted line) were computed for each list and corrected for multiple comparisons (*P < 0.05). **(B)** Confidence score on a 3-level scale (null, low, high) for memory response of the NREM list. Statistical tests reveal no difference between trials that were successfully identified (correct) and those associated with a mistake (error).

We first investigated whether participants learned the meaning of words presented during sleep. We first observed that memory performance depended on arousal level [repeated-measure ANOVA, F(2,21) = 4.66, *p* = 0.021]. This effect came from a higher memory performance for words from the NREM list compared to words that were not presented during sleep, i.e., the control list (*post hoc* Tukey-test, NREM vs. control: *p* = 0.011; REM vs. control, *p* = 0.201; NREM vs. REM, *p* = 0.407). Memory was above chance level for words from the NREM list [59%, CI = (52, 66)], Cohen’s d = 0.58, two-tailed Student’s t -test against chance level [50%, t(21) = 2.7, p = 0.013, corrected for false discovery rate (Benjamini and Yekutieli, 2001; Figure 2A)]. Comparatively, memory was not different from the chance level for words from the REM list [53%, CI = (48, 58), *d* = 0.28, two-tailed Student’s *t*-test against chance level, t(21) = 1.32, *p* = 0.201]. Memory for words from the list that was not presented during sleep, i.e., the control list, also did not differ from the chance level, showing that learning could not be attributed to prior knowledge of the meaning of Japanese words [45%, CI = (38, 53), *d* = −0.27, two-tailed Student’s *t*-test against chance level, t(21) = −1.31, *p* = 0.204; Figure 2A].

We then checked whether sleep learning could be attributed to items being more easily acquired over others. We thus fitted memory performance at the single item level (generalized linear mixed model with binomial distribution (correct vs. incorrect), participants as random variable and list as a covariate). Memory performance depended on lists [analysis of variance: F(2,1053) = 6.31, *p* = 0.002]. We thus focused on the NREM list and found no influence of item identity on our results [analysis of variance: F(47,304) = 0.93]. These analyses show that memory performance could not be attributed to the learning of the same specific items across participants.

We further checked that memory for the NREM list was not due to items that were inadvertently played during awakenings or micro-arousals [3.4 items per subject, CI = (2.0, 4.8), see Supplementary Table 2]. We observed that the effect of memory for the NREM list was significant even after removing these items [58%, CI = (50, 68), *d* = 0.45, one-tailed Student’s *t*-test against chance level, t(20) = 2.04, *p* = 0.028]. These results confirm that the meaning of Japanese words was acquired during NREM sleep.

### Implicit Memory

We then investigated whether the nature of the memory formed during NREM sleep was implicit or explicit. If learning during NREM sleep was implicit, we expected that confidence scores would be similar for words from the NREM list vs. words from the control list, and for correct vs. incorrect memory choices. Consistent with this hypothesis, we found no difference in confidence scores between the NREM list and the control list [−0.03, CI = (−0.11, 0.05), *d* = −0.01, paired two-tailed Student’s *t*-test for the difference between the NREM and control list, t(21) = −0.78, *p* = 0.444]. Confidence scores were also similar between items of the NREM list that were correctly identified (correct) and items that were incorrect (error) [0.00, CI = (−0.12, 0.12), *d* = 0.01, paired two-tailed Student’s *t*-test for the difference between correct and error, t(21) = 0.05, *p* = 0.959; Figure 2B]. These results thus demonstrate that memory formation during NREM sleep is implicit.

We also found no difference in confidence scores between words from the REM and control list [−0.01, CI = (−0.08, 0.07), *d* = −0.03, paired two-tailed Student’s *t*-test for the difference between the REM and control list, t(21) = −0.16, *p* = 0.877], as well as between correct and error responses for the REM list [0.08, CI = (−0.02, 0.20), *d* = 0.34, paired two-tailed Student’s *t*-test for the difference between correct and error trials, t(21) = 1.63, *p* = 0.117] and for the control list [0.10, CI = (−0.05 0.25), *d* = 0.30, paired two-tailed Student’s *t*-test for the difference between correct and error trials, t(21) = 1.39, *p* = 0.180]. Confidence scores thus confirm the absence of evidence for learning for both the REM and control lists.

We finally ran a control experiment during which new participants performed the experiment entirely during wakefulness (*n* = 12). Participants acquired the meaning of Japanese words heard during wakefulness, and importantly, higher confidence scores were found for items learned during wakefulness as compared to the control list, as well as for correctly identified items as compared to incorrect ones (Supplementary Figure 1). These results show that implicit memory formation observed during sleep qualitatively differs from explicit memory formation obtained during wakefulness.

### Neural Signatures of Sleep Learning

To understand the neural processes supporting memory formation during sleep, we investigated whether memory performance after awakening could be predicted by the brain responses to stimuli played during NREM sleep. To do so, we contrasted the brain responses to stimuli that were correctly identified during the memory test and those associated with an error. We hypothesized that stimulus presentation triggered the entrainment of neural oscillation in the slow-wave (SW) range, a well-documented phenomenon happening during NREM sleep (Bastien and Campbell, 1992; Halász, 2016). We thus selected for our analyses a region-of-interest composed of a frontal cluster of electrodes where slow-waves have the strongest amplitude (Massimini et al., 2004) and where memory effects have been reported in previous literature (Züst et al., 2019).

**FIGURE 3:**
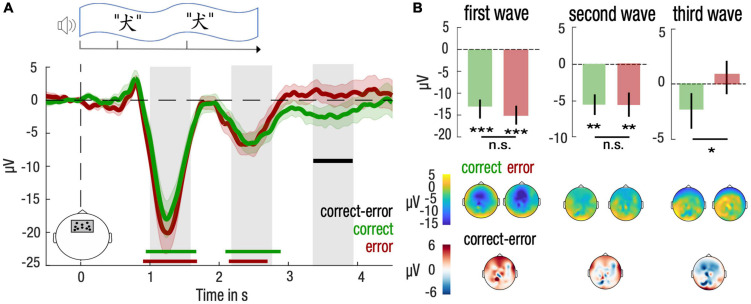
Differential entrainment of frontal neural responses at a slow-wave frequency to stimulation for correctly and incorrectly identified items during NREM sleep. **(A)** Neural responses were computed over a frontal cluster of electrodes depicted in the lower left panel. Time-course of the stimulus presentation was indicated in a top panel. Time-courses of the brain responses (lower panel) were averaged across participants and smoothed for visualization purposes only, using a 500-ms wide Gaussian kernel. Mean and standard error of the mean (SEM) are represented, respectively, with solid lines and shaded areas for the correct (green) and the error (red) trials. Statistical tests were performed on brain responses before smoothing. Green, red and black horizontal lines denote significant clusters of neural responses differing from baseline (0, dotted line) across participants for, respectively, correct trials, error trials, or the difference between both conditions (*P* < 0.05 after cluster correction). Gray bars indicate time clusters corresponding to negative half-periods of a slow wave rhythm at 0.85 Hz. **(B)** Neural responses were averaged over each negative half-period of slow waves and compared between correct (green) and error (red) trials. Mean and standard error of the mean (SEM) are represented, respectively, with bar plots and solid lines. Student’s *T*-test against baseline (0, dotted line) was computed for correct and error trials and corrected for multiple comparisons. Paired Student’s *T*-test were computed between correct and error trials (***P < 0.001, **P < 0.01, *P < 0.05). Topography of amplitudes were indicated in lower panels for correct, error and the difference between correct and error trials.

For both correct and error trials, we obtained an expected modulation of brain responses after the presentation of the first and second word [correct vs. baseline: (0.94, 1.67)s, *d* = −0.54, Monte-Carlo test, alpha-level = 0.05, 1,000 permutations, Σt(21) = −353.7, P_cluster_ = 0.006 and (2.09, 2.89)s, *d* = −0.40, Σt(21) = −353.7, P_cluster_ = 0.009; error vs. baseline: (0.90, 1.68)s, *d* = −0.85, Σt(21) = −414.9, P_cluster_ < 0.001 and (2.14, 2.70)s, *d* = −0.37, Σt(21) = −192.7, P_cluster_ = 0.030 after cluster correction; Figure 3A]. In addition, we observed a significant difference between correct and error trials only after the end of the stimulation [correct vs. error: (3.36, 3.93)s, *d* = −0.44, Σt(21) = −120.1, P_cluster_ = 0.029]. We thus investigated in more details the amplitude of brain responses corresponding to the human SW range (0.85 Hz) (gray bars, Figure 3A).

*Post hoc* tests confirmed that amplitudes differed significantly from baseline both for correct and error trials at the 1^st^ and 2^nd^ SW (Student’s *t*-test against baseline (0), all *p*-values inferior to 0.05, corrected for multiple comparisons; Figure 3B). Brain topography of these responses confirmed that amplitudes were maximal over frontal electrodes. *Post hoc* results did not reveal any difference between correct and error trials for the 1^st^ and 2^nd^ SW (paired two-tailed Student’s *t*-test for correct vs. error trials, *P* > 0.05 for the 1^st^ and 2^nd^ SW; Figure 3B). However, for the 3^rd^ SW, *post hoc* comparisons confirmed a lower amplitude for correct trials compared to error trials [−3.22 μV, CI = (−6.3, −0.2), *d* = −0.46, t(21) = −2.2, *p* = 0.039, corrected for multiple comparison; Figure 3B].

### Dynamics of Sleep Learning

**FIGURE 4:**
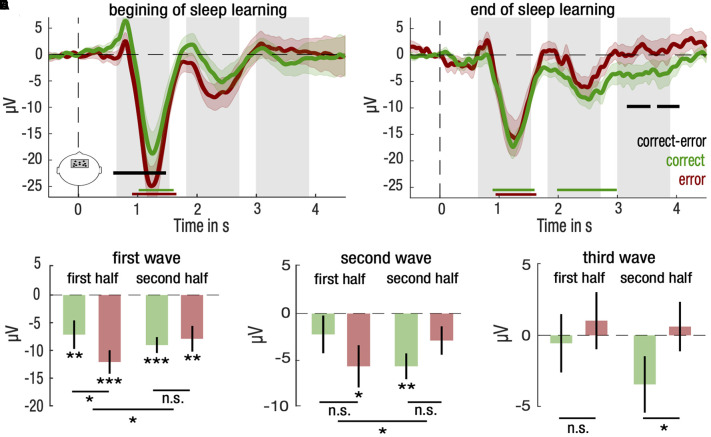
Differential entrainment of frontal neural responses to stimulation for correctly and incorrectly identified items for the first half and the second half of NREM trials. **(A,B)** Mean and standard error of the mean (SEM) are represented, respectively, with solid lines and shaded areas for the correct (green) and the error (red) trials for the first **(A)** and second **(B)** half trials in NREM sleep. Green, red and black horizontal lines denote significant clusters of neural responses differing from baseline (0, dotted line) for, respectively, correct trials, error trials, or the difference between both conditions (*P* < 0.05 after cluster correction). Gray bars indicate time clusters spanning over the three-fourth of a slow wave rhythm at 0.85 Hz centered over its trough. **(C–E)** Neural responses for correct (green) and error (red) trials for the first and second half of trials were averaged and compared for the first **(C)**, second **(D)** and third **(E)** slow wave. Mean and standard error of the mean (SEM) are represented, respectively, with bar plots and solid lines. Student’s *T*-test against baseline (0, dotted line) was computed for correct and error trials and corrected for multiple comparisons. Paired Student’s *T*-test were computed between correct and error trials for and corrected for multiple comparisons. Interaction between trial type (correct vs. error) and period of the night (first vs. second half) for each slow wave was computed with repeated measures ANOVA (***P < 0.001, **P < 0.01, *P < 0.05).

Finally, we investigated whether we could track the learning process during sleep. To do so, we split the sleep learning phase in two equal parts and compared brain responses to stimuli for correct and error trials in the 1^st^ and 2^nd^ half of the sleep learning phase. For the 1^st^ half, we found a positive cluster covering the 1^st^ SW [correct vs. error: (0.59, 1.48)s, *d* = −0.49, Σt(21) = 205.0, P_cluster_ = 0.025; Figure 4A]. For the 2^nd^ half, two late clusters emerged over the 3^rd^ SW [correct vs. error: (3.16, 3.56)s, *d* = −0.46, Σt(21) = −88.9, P_cluster_ = 0.047, and (3.67, 4.05)s, *d* = −0.49, Σt(21) = −90.0, P_cluster_ = 0.046; Figure 4B]. As such, we investigated separately for each SW period whether the type of trials, i.e., correct vs. error, interacted with the part of the night, i.e., 1^st^ vs. 2^nd^ half.

We found such an interaction for brain amplitudes of the 1^st^ SW and the 2^nd^ SW periods (repeated measures ANOVA; 1^st^ SW: F(1,21) = 4.52, *p* = 0.046, 2^nd^ SW: F(1,21) = 4.45, *p* = 0.047; Figures 4C,D). *Post hoc* analyses revealed that for the 1^st^ SW, brain differences between correct and error trials were restricted to the 1^st^ half, suggesting that the 1^st^ SW reflected differences relative to the initial encoding of stimuli [4.9 μV, CI = (1.1, 8.7), *d* = 0.48, paired two-tailed Student’s *t*-test for correct vs. error trials, t(21) = 2.68, *p* = 0.028, corrected for multiple comparison; Figure 4C]. For the 2^nd^ SW, the pattern of response reversed from 1^st^ to the 2^nd^ half of the night, with brain potentials lower than baseline for error trials in the 1^st^ half and for correct trials in the 2^nd^ half [1^st^ half error: −5.7 μV, CI = (−10.3, −1.1), *d* = −0.51, two-tailed Student’s *t*-test against baseline (0), t(21) = −2.56, *p* = 0.037; 2^nd^ half correct: 5.7 μV, CI = (−8.5, −2.8), *d* = −0.89, two-tailed Student’s *t*-test against baseline (0), t(21) = 4.19, *p* = 0.002, corrected for multiple comparison; Figure 4D].

Despite the fact that no interaction was observed at the 3^rd^ SW (repeated measures ANOVA; F(1,21) = 1.06, *p* = 0.315), *post hoc* tests showed a difference in responses restricted to the 2^nd^ half of the night [−4.2 μV, CI = (−7.7, −0.7), *d* = −0.48, paired two-tailed Student’s *t*-test for correct vs. error trials, t(21) = −2.46, *p* = 0.046, corrected for multiple comparison; Figure 4E]. Overall, these results show that neural responses predicting successful memory retrieval shift from early evoked SW activity to late evoked SW activity throughout the course of the sleep learning phase.

## Discussion

Even while asleep, the brain remains able to process its sensory environment (Andrillon and Kouider, 2020). The sleeping brain can discriminate complex sound properties (Bastuji et al., 1995; Kouider et al., 2014; Strauss et al., 2015; Blume et al., 2017), and even learn perceptual information (Ruch et al., 2014; Andrillon and Kouider, 2016; Andrillon et al., 2017) and novel sensory associations (Arzi et al., 2012, 2014; Züst et al., 2019). Yet, the extent to which sensory associations acquired during sleep can be flexibly retrieved after awakening remains poorly understood (Ruch and Henke, 2020).

Here, we demonstrate that new associations between unknown words and sensory representations referring to their meaning can be formed during sleep and retrieved across sensory modalities during wakefulness. We obtained remarkably similar effect sizes as a recent study showing the formation of new verbal associations during sleep (59% vs. 55%, Züst et al., 2019), replicating the finding that new vocabulary can be acquired during sleep. However, by relying on a cross-modal identification task (e.g., “does this word correspond to the picture of a dog or a picture of a bell?”) rather than a categorization task (e.g., “is the size of item bigger or smaller than a shoebox?”), our results provide first evidence that new associations can be transferred from sleep to wakefulness and retrieved cross-modally.

We also provide evidence that the memory formed during sleep is retrieved implicitly, meaning that participants remained unaware of their newly acquired knowledge. While learning during sleep has often been deemed implicit, studies rarely tested this assumption directly [e.g., Wood et al., 1992; Ruch et al., 2014; Züst et al., 2019; see Andrillon and Kouider (2016) for an exception]. Here, we asked participants to report their confidence after each memory decision and compared second-order responses, i.e., confidence score, as a function of first-order responses, i.e., correct or incorrect memory responses, to probe the implicit nature of memory formed during sleep (Dienes and Berry, 1997; Yeung and Summerfield, 2012; Andrillon and Kouider, 2016). We found that confidence scores did not differ between correct and incorrect memory decisions when the learning was performed during sleep. On the contrary, when learning was performed during wakefulness in a control experiment, we obtained higher confidence scores for correct vs. incorrect decisions. This finding clearly distinguishes between explicit learning observed during wakefulness and implicit learning occurring during sleep.

Our results thus confirm that cross-modal associations can be transferred implicitly (Berry et al., 1997; Blum and Yonelinas, 2001; Scott et al., 2018). Contrary to previous studies that relied on subliminal stimuli to test for this possibility, we demonstrate here such implicit transfer by altering participants’ conscious level rather than degrading stimulus properties during memory formation (Kouider and Dehaene, 2007). Additionally, our novel Associative Transfer Learning (ATL) relied on presenting separately the two pieces of information that were tested after awakening, i.e., the image and its corresponding sound during wakefulness and the Japanese translation and the same corresponding sound during sleep. By showing that new associations can be transferred beyond the sensory format in which they were presented, we demonstrated that associative learning, when transferred across conscious states, can be generalized across sensory modalities.

A remaining question concerns the exact origin of the cross-modal generalization that we observed in this study, and whether it results from cross-modal retrieval at awakening or cross-modal encoding during sleep. In the former case, Japanese words and their corresponding sound would be associated during sleep, and images would then be retrieved after awakening upon presentation of the Japanese word. In the latter case, ATL would consist in the reactivation of images upon their sound presentation and the formation of a cross-modal mapping between the Japanese word and the image during sleep. Presenting sounds during sleep is known indeed to trigger the reactivation and memory consolidation of memory traces associated with the sound, a technique called Targeted Memory Reactivation (Rasch et al., 2007; Oudiette and Paller, 2013). Our results suggest additionally that reactivating memory traces may lead to the formation of new cross-modal associations between during sleep. Further studies should elucidate the nature of the transfer of cross-modal associations across conscious states.

We then investigated the neural basis of memory formation during sleep to further elucidate the mechanisms involved in ATL. Frontal activity at a slow-wave frequency (0.85 Hz), a hallmark of NREM sleep, was evoked by stimulus presentation during sleep and predicted memory performance after awakening. Our study is in line in this respect with previous results showing that learning during sleep is bound to slow-wave activity and thus reinforces the view that slow waves play an active role in memory processes during sleep (Marshall et al., 2006; Klinzing et al., 2019; Züst et al., 2019; Canales-Johnson et al., 2020). While Züst et al. (2019) obtained similar results during deep NREM sleep, we provide evidence that these results also hold true for light NREM sleep (Supplementary Figure 2 and Supplementary Table 3). We also provide direct evidence that the neural markers predictive of associative memory formation evolve during sleep, allowing us to track the learning process.

In the beginning of the sleep learning process, SW responses were enhanced during the first wave triggered by stimulus onset and could reflect differences in the initial encoding of sensory information (Dang-Vu et al., 2011). At the end of the sleep learning process, SW responses were stronger during the third wave after stimulus offset. This late pattern of response could reflect the entrainment of frontal responses by reverberating processing in associative networks built over the course of learning (Cox et al., 2014). Could these neural signals also result from the spontaneous changes in neural activity observed during sleep and not only reflect the sleep learning process? Contrary to our observations, spontaneous SW activity during NREM sleep tends to decrease over the course of the night (Nir et al., 2011). Thus, the dynamics of neural signatures of learning cannot solely be explained by changes in the sleep structure.

We further investigated whether ATL depended on the distinct neural states of sleep, notably non-Rapid Eye Movement (NREM) and Rapid Eye Movement (REM) sleep. Studies of sensory conditioning during sleep revealed that sound-odor associations were transferred into wakefulness when played during NREM sleep but not during REM sleep (Arzi et al., 2012, 2014). Using a very different task and stimulation procedure, our results converge into showing that ATL is observed in NREM sleep and not REM sleep. A potential explanation for this fact could be that dreaming activity, enriched during REM sleep as compared to NREM sleep, might compete for attentional resources with the processing of external information and prevent the encoding of new information during REM sleep (Nir and Tononi, 2010; Andrillon et al., 2016; Andrillon and Kouider, 2020). However, an alternative explanation may be the lower number of presentations during REM sleep as compared to NREM sleep obtained in our study (3 times less, 13 vs. 40) (Supplementary Table 2), preventing any direct comparison to be made about sleep learning across sleep states based on these results. Further experiments could focus on REM sleep to investigate whether ATL can be observed during REM sleep.

Finally, our study did not provide an explanation as to why certain items triggered sleep learning and their associated neural correlates while others did not, as well as why some participants obtained higher performance than others. Items were selected for being easily heard and recognized, and stimuli were furthermore carefully matched in terms of intensity and duration. Accordingly, no single item was found to be more easily learnt across participants, confirming that acoustic features play a limited role in explaining observed differences. Individual preferences for certain items may explain how different items were more easily acquired across participants. Additionally, general ability to speak and learn languages, as well as traits like memory capacity and motivation, could also explain interindividual differences in sleep learning abilities. While these factors cannot account for our findings about cross-modal identification of new words acquired during NREM sleep, it might be of interest for future studies to determine the contribution of these factors to sleep learning.

Overall, our results demonstrate that new memory associations acquired during sleep can be transferred implicitly and generalized across modalities into wakefulness. In line with previous studies, we showed that slow-wave activity following stimulus presentation during NREM sleep predicted memory formation (Züst et al., 2019; Canales-Johnson et al., 2020). We further demonstrate that this neural marker evolves over the course of the learning process, providing direct evidence that slow-wave activity tracks the formation and evolution of associative memory traces during NREM sleep. It is also noteworthy that explicit learning during wakefulness is still much more efficient compared to implicit learning during sleep, since ten times less repetition (4 against 40) were sufficient to achieve considerably higher accuracy (88% vs. 59%) while participants were aware which are the words for which they have successfully acquired the meaning. It remains unclear to which extent cross-modal generalization of new memory traces during ATL occurs at encoding during sleep or at retrieval during wakefulness and whether ATL can be extended to REM sleep, and further experiments are required to settle these questions.

## Data Availability Statement

The raw data supporting the conclusions of this article will be made available by the authors, without undue reservation.

## Ethics Statement

The studies involving human participants were reviewed and approved by Comité de Protection des Personnes, Ile-de-France I, Paris, France. The patients/participants provided their written informed consent to participate in this study.

## Author Contributions

MK and SK developed the study concept and design. MK performed testing and data collection with the help of ME and DL. MK performed the data analysis and interpretation under the supervision of DL and SK. MK, DL, and SK wrote the manuscript. All authors approved the final version of the manuscript for submission.

## Conflict of Interest

The authors declare that the research was conducted in the absence of any commercial or financial relationships that could be construed as a potential conflict of interest.

## Publisher’s Note

All claims expressed in this article are solely those of the authors and do not necessarily represent those of their affiliated organizations, or those of the publisher, the editors and the reviewers. Any product that may be evaluated in this article, or claim that may be made by its manufacturer, is not guaranteed or endorsed by the publisher.

## Footnotes

1
https://ttsmp3.com/

2
https://eng.universal-soundbank.com

3
https://www.freesoundeffects.com/
