# Supplementary material for: Learning New Vocabulary Implicitly During Sleep Transfers With Cross-Modal Generalization Into Wakefulness
Source: Front Neurosci. 2022 Mar 9;16:801666. doi: 10.3389/fnins.2022.801666 (PMC8959773; doi:10.3389/fnins.2022.801666)
Supplement: Supplementary file 1 [file Data_Sheet_1.pdf]

Supplementary Material

An absence of difference in confidence scores in our main experiments could arise from the inability of participants to correctly use the confidence scale. To rule out this hypothesis, we ran a control experiment with new participants during wakefulness (N=12, Figure S1). The stimuli and task were the same as in our main experiment except that the learning phase was performed awake. During this phase, twenty-four associations (wake list) were repeated four times and twenty-four associations were not presented (control list). We found that memory performance for the wake list was higher than chance (88%, CI=[80, 97]) and higher than for the control list (Student's t-test between the wake and control list, $t(11)=7.1$, $p<0.001$; Figure S1). The control list did not differ from chance level (52%, CI=[45, 59]; $t(11)=0.68$, $p=0.510$). We then investigate confidence scores to test whether we could find evidence for explicit memory formation during wakefulness. Confidence scores for the wake list were higher than those observed for the control list (2.8, CI=[2.7, 2.9] *vs.* 1.8, CI=[1.6, 2.0], Student's t-test between wake and control list, $t(11)=7.1$, $p<0.001$). Additionally, confidence score for correct trials were higher than incorrect only for the wake list (repeated measures ANOVA; $F(1,8)=10.91$, $p=0.0108$; post-hoc Student's t-test correct *vs.* incorrect for the wake list, $t(8)=4.43$, $p=0.002$; Figure S1). These results confirm that our measures of confidence scores allow us to unravel the differences between explicit and implicit learning occurring respectively during wakefulness and sleep.

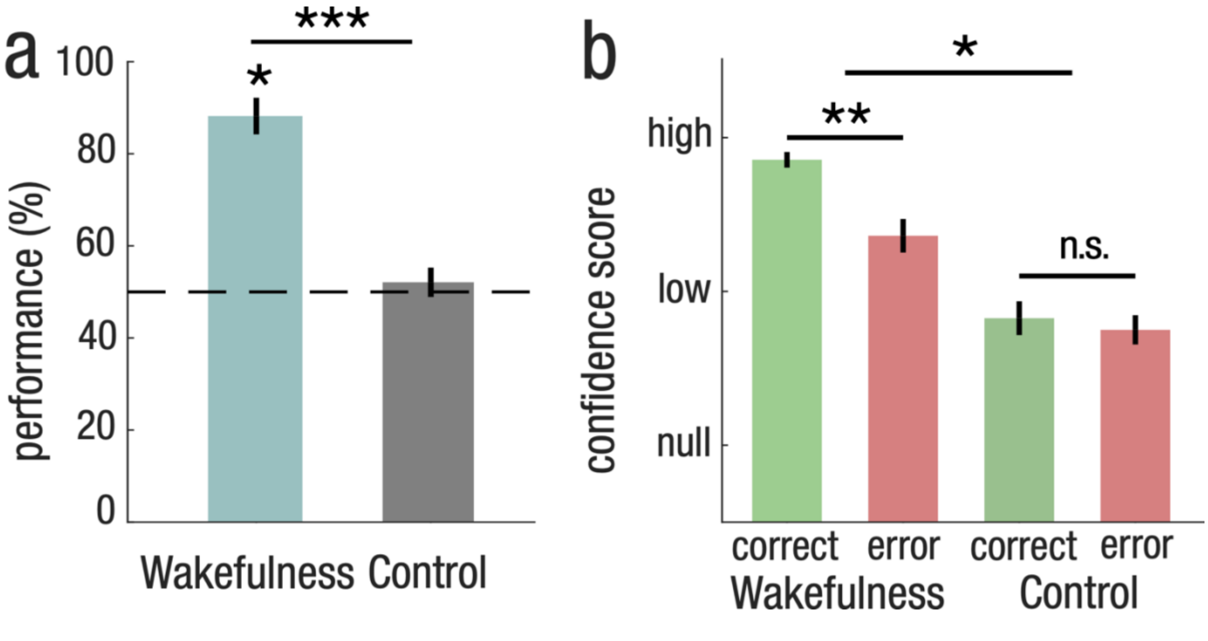

Figure S1. **Explicit memory formation during wakefulness**
(**A**) Memory performance for wake (24 items) and Control (24 items) list. This experiment followed the same structure as the sleep experiment, except the sleep learning phase was performed during wakefulness and associations were repeated 4 times.
(**B**) Confidence score for the wake and error list. Interaction between memory response (correct *vs.* error) and list (wake *vs.* control) was tested with repeated-measure ANOVA with participants as a random factor. Difference across conditions were calculated with paired Student's T-test (***, P<0.001; **, P<0.01; *<0.05).

In our main experiment, we tracked the sleep learning process using frontal slow-wave activity in response to sounds. Because deep NREM is characterized by a stronger slow-wave (SW) activity than light NREM sleep, we checked whether differences observed between correct *vs.* incorrect items could not be only explained by trials in deep NREM sleep. To test this hypothesis, we restricted our analysis to trials belonging to light NREM sleep (484 trials per participant, CI=[401, 568], see Table S1). We observed that our results remain and even observed a larger cluster of difference between correct and error trials, spanning over the whole 3rd SW ([2.76, 3.89]s, $t(21)=-248.7$, $P_{cluster}=0.017$, corrected for cluster comparison; Figure S2A). Post-hoc tests confirmed that our described effects from the whole NREM sleep period are conserved when restricting our results to light NREM sleep (Figure S2B). This analysis thus confirms that the results observed for the whole NREM sleep period also hold for light NREM sleep. Because our experiment was run at the end of the night, the same analyses restricted to deep NREM could not be conducted due to the low and unequal duration of deep NREM sleep that we obtained across participants (23±4.7 minutes *vs.* 102±5.7 in light NREM sleep, see Table S3 for full sleep statistics).

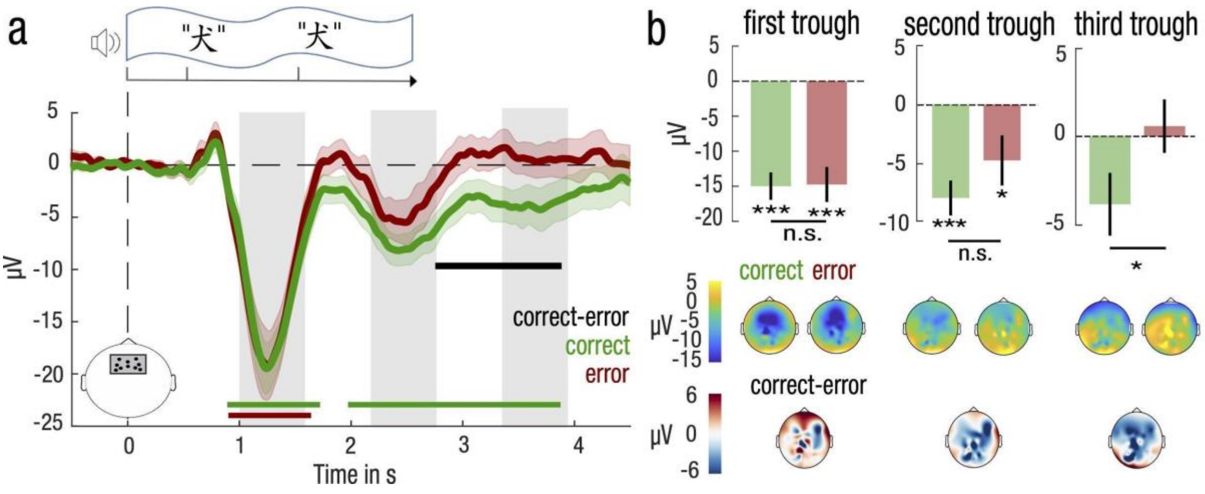

Figure S2. **Entrainment of frontal neural responses at a slow-wave frequency by stimulus presentation for correctly and incorrectly identified items during light NREM sleep** (**A**) Neural responses were computed over a frontal cluster of electrodes depicted in the lower left panel. Time-course of the stimulus presentation was indicated in a top panel. Neural responses were here restricted to NREM trials that were scored as part of light NREM sleep (NREM2). Mean and standard error of the mean (SEM) are represented respectively with solid lines and shaded areas for the correct (green) and the error (red) trials. Green and red horizontal lines denote significant clusters of neural responses that differ from baseline (0, dotted line) for respectively correct and error trials. Grey bars indicate time clusters corresponding to negative half-periods of a slow wave rhythm at 0.85Hz.

(**B**) Neural responses were averaged over each negative half-period of slow waves and compared between correct (green) and error (red) trials. Mean and standard error of the mean (SEM) are represented respectively with bar plots and solid lines. Student's T-test against baseline (0, dotted line) was computed for correct and error trials and corrected for multiple comparisons. Paired Student's T-test were computed between correct and error trials (***: $P<0.001$, **: $P<0.01$, *: $P<0.05$). Topography of amplitudes were indicated in lower panels for correct, error and the difference between correct and error.

4.7 (CI=[2.9, 6.7]) items of the NREM list were played once over 40 times during brief periods of (micro-)arousal during the sleep learning phase. To ensure that our EEG responses were not driven by these items played during brief periods of awakening, we performed the same analyses after having removed these items from all our EEG analyses.

For both correct and error trials, we obtained an expected modulation of brain responses after the presentation of the first and second word (correct *vs.* baseline: [0.99, 1.60]s, d=-0.53, Monte-Carlo test, alpha-level=0.05, 1000 permutations, $\sum t(21)=-252.2$, $P_{cluster}=.009$ and [2.23, 2.82]s, d=-0.43, $\sum t(21)=-144.0$, $P_{cluster}=.046$; error vs. baseline: [0.91, 1.69]s, d=-0.72, $\sum t(21)=-414.9$, $P_{cluster}=.001$ and [2.16, 2.70]s, d=-0.32, $\sum t(21)=-158.8$, $P_{cluster}=.036$ after cluster correction; Figure S3A). However, we observed a significant difference between correct and error trials only after the end of the stimulation (correct *vs.* error: [3.17, 3.81]s, d=-0.39, $\sum t(21)=-153.1$, $P_{cluster}=.025$).

Post-hoc tests confirmed that amplitudes differed significantly from baseline both for correct and error trials at the 1st and 2nd SW (Student's t-test against baseline (0), all p-values inferior to 0.05, corrected for multiple comparisons; Figure S3B). Brain topography of these responses confirmed that amplitudes were maximal over frontal electrodes. Post-hoc results did not reveal any difference between correct and error trials for the 1st and 2nd SW (paired two-tailed Student's t-test for correct *vs.* error trials, P>.05 for the 1st and 2nd SW; Figure S3B). Yet, for the 3rd SW, post-hoc comparisons confirmed a lower amplitude for correct trials compared to error trials (-3.75 μV, CI=[-6.6, -0.9], d=-0.63, t(18)=-2.8, p=.012, corrected for multiple comparison; Figure S3B).

After restricting our analysis to trials belonging to light NREM sleep, we observed that our results remain and even observed a larger cluster of difference between correct and error trials, spanning over the whole 3rd SW (correct vs. error: [3.16, 3.95]s, d=-0.53, t(21)=-182.3, $P_{cluster}=0.034$, corrected for cluster comparison; Figure S4A). Post-hoc tests confirmed that our described effects from the whole NREM sleep period are conserved when restricting our results to light NREM sleep (Figure S4B). This analysis thus confirms that the results observed for the whole NREM sleep period also hold for light NREM sleep even after rejecting trials of items that have been presented during brief periods of awakening.

Finally, we investigated whether we could track the learning process during sleep. To do so, we split the sleep learning phase in two equal parts and compared brain responses to stimuli for correct and error trials in the 1st and 2nd half of the sleep learning phase. The effects were similar to our main results even if they did not reach significance due to a lower amount of trials and thus noisier EEG signals than in the main analyses (Figure S5).

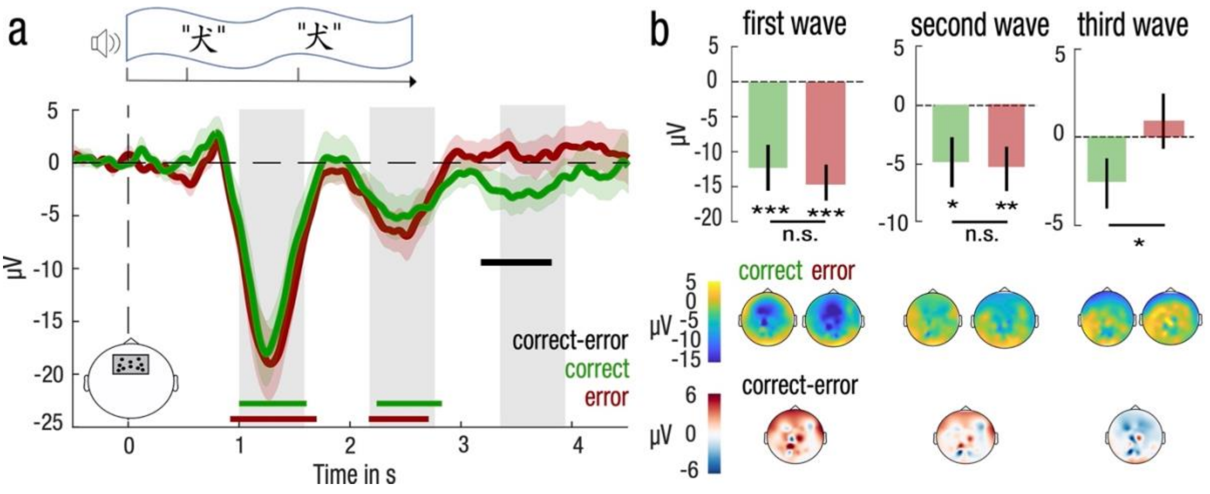

Figure S3. **Differential entrainment of frontal neural responses at a slow-wave frequency to stimulation for correctly and incorrectly identified items during NREM sleep after removal of items that have been presented during (micro-)awakenings.**
(**A**) Neural responses were computed over a frontal cluster of electrodes depicted in the lower left panel. Time-course of the stimulus presentation was indicated in a top panel. Time-courses of the brain responses (lower panel) were averaged across participants and smoothed for visualization purposes only, using a 500-ms wide Gaussian kernel. Mean and standard error of the mean (SEM) are represented respectively with solid lines and shaded areas for the correct (green) and the error (red) trials. Statistical tests were performed on brain responses before smoothing. Green, red and black horizontal lines denote significant clusters of neural responses differing from baseline (0, dotted line) across participants for respectively correct trials, error trials, or the difference between both conditions (P<.05 after cluster correction). Grey bars indicate time clusters corresponding to negative half-periods of a slow wave rhythm at 0.85Hz.
(**B**) Neural responses were averaged over each negative half-period of slow waves and compared between correct (green) and error (red) trials. Mean and standard error of the mean (SEM) are represented respectively with bar plots and solid lines. Student's T-test against baseline (0, dotted line) was computed for correct and error trials and corrected for multiple comparisons. Paired Student's T-test were computed between correct and error trials (***: P<.001, **: P<.01, *: P<.05). Topography of amplitudes were indicated in lower panels for correct, error and the difference between correct and error trials.

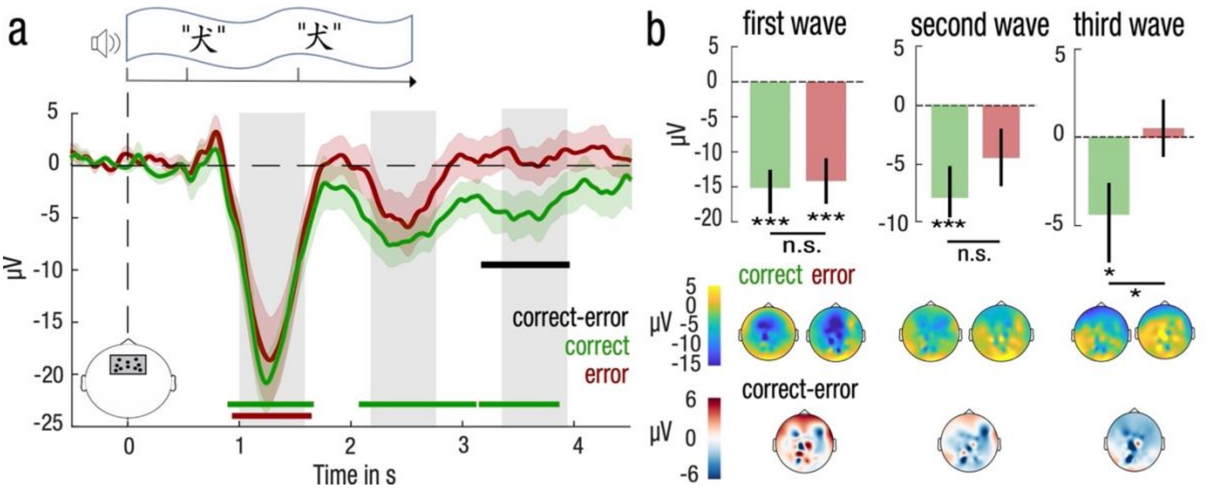

Figure S4. **Entrainment of frontal neural responses at a slow-wave frequency by stimulus presentation for correctly and incorrectly identified items during light NREM sleep after removal of items that have been presented during (micro-)awakenings.**

(**A**) Neural responses were computed over a frontal cluster of electrodes depicted in the lower left panel. Time-course of the stimulus presentation was indicated in a top panel. Neural responses were here restricted to NREM trials that were scored as part of light NREM sleep (NREM2). Mean and standard error of the mean (SEM) are represented respectively with solid lines and shaded areas for the correct (green) and the error (red) trials. Green and red horizontal lines denote significant clusters of neural responses that differ from baseline (0, dotted line) for respectively correct and error trials. Grey bars indicate time clusters corresponding to negative half-periods of a slow wave rhythm at 0.85Hz.

(**B**) Neural responses were averaged over each negative half-period of slow waves and compared between correct (green) and error (red) trials. Mean and standard error of the mean (SEM) are represented respectively with bar plots and solid lines. Student's T-test against baseline (0, dotted line) was computed for correct and error trials and corrected for multiple comparisons. Paired Student's T-test were computed between correct and error trials (***: P<0.001, **: P<0.01, *: P<0.05). Topography of amplitudes were indicated in lower panels for correct, error and the difference between correct and error.

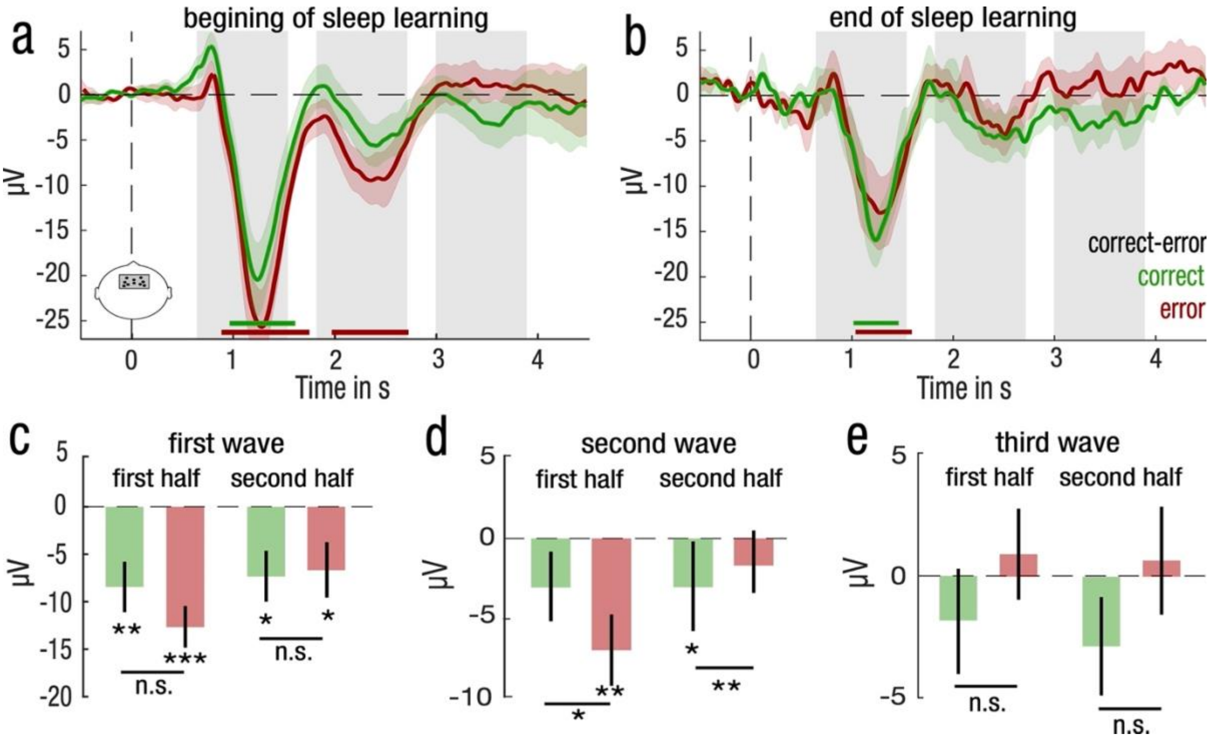

Figure S5. **Differential entrainment of frontal neural responses to stimulation for correctly and incorrectly identified items for the first half and the second half of NREM trials after removal of items that have been presented during (micro-)awakenings.**
(**A,B**) Mean and standard error of the mean (SEM) are represented respectively with solid lines and shaded areas for the correct (green) and the error (red) trials for the first (A) and second (B) half trials in NREM sleep. Green, red and black horizontal lines denote significant clusters of neural responses differing from baseline (0, dotted line) for respectively correct trials, error trials, or the difference between both conditions (P<.05 after cluster correction). Grey bars indicate time clusters spanning over the three-fourth of a slow wave rhythm at 0.85Hz centered over its trough.
(**C-E**) Neural responses for correct (green) and error (red) trials for the first and second half of trials were averaged and compared for the first (C), second (D) and third (E) slow wave. Mean and standard error of the mean (SEM) are represented respectively with bar plots and solid lines. Student's T-test against baseline (0, dotted line) was computed for correct and error trials and corrected for multiple comparisons. Paired Student's T-test were computed between correct and error trials for and corrected for multiple comparisons. Interaction between trial type (correct *vs.* error) and period of the night (first *vs.* second half) for each slow wave was computed with repeated measures ANOVA (***: P<.001, **: P<.01, *: P<.05).

In our main analyses, we hypothesized that memory decision was based on the identification of the Japanese word presented during the 2-Alternative Forced Choice (2AFC) test. Thus, we defined correct and error trials during sleep learning according to whether the Japanese word belonged to a correct or error trial during the 2AFC. Nevertheless, a correct response in the 2AFC could result from two different evaluations: either the identification of the picture corresponding to the Japanese word (referred hereafter as image A) or identification of the other picture that was presented in the same trial and that did not correspond to the Japanese word (referred hereafter as image B). Thus, a correct response might not necessarily stem from learning the presented word. We therefore verified the contribution of image B to the memory decision by checking how including them in the definition of correct *vs.* error trials impacted the neural correlates of sleep learning. We conducted three analyses:

First, trials were defined as correct for items corresponding to image A and to image B in a correct memory decision, irrespective of whether image B was correctly identified in its own trial. We found expected modulations of the brain responses at a slow-wave rhythm (correct *vs.* baseline: [0.97, 1.60]s, d=-0.54, Monte-Carlo test, alpha-level=0.05, 1000 permutations, $\sum t(21)=-342.5$, $P_{cluster}=.002$ and [2.18, 2.77]s, d=-0.09, $\sum t(21)=-180.5$, $P_{cluster}=.033$; error *vs.* baseline: [0.88, 1.64]s, d=-0.59, $\sum t(21)=-361.8$, $P_{cluster}<.001$ and [2.94, 3.90]s, d=0.67, $\sum t(21)=247.4$, $P_{cluster}=.006$), as well as a difference between correct and error trials for the first and third wave (correct *vs.* error: [0.34, 1.03]s, d=0.23, $\sum t(21)=160.77$, $P_{cluster}=.035$ and [2.99, 4.03]s, d=-0.15, $\sum t(21)=-238.3$, $P_{cluster}=.007$; Figure S6A). This result is in line with our main results combining the beginning and the end of the sleep learning phase. One interpretation of this result is that it reflects the presence of noise in the 2AFC decision process. By including both image A and image B as correct trials, learned items can be counted as correct despite incorrect decisions were made when they were presented as image A. Alternatively, another interpretation of this result is that it reflects the contribution of image B to memory decision.

To test for the second hypothesis, trials were defined as correct for items corresponding to image B in a correct memory decision. This contrast allows us to check whether a memory decision based on the identification of image B is sufficient to account for the EEG differences between correct and error trials during sleep learning. We found an expected modulation of the frontal response for the first and second waves (correct *vs.* baseline: [0.97, 1.59]s, d=-0.56, $\sum t(21)=-282.0$, $P_{cluster}=.009$ and [2.23, 2.82]s, d=-0.53, $\sum t(21)=-175.4$, $P_{cluster}=.034$; error *vs.* baseline: [0.88, 1.74]s, d=-0.88, $\sum t(21)=-447.6$, $P_{cluster}=.004$ and [1.98, 2.79]s, d=-0.65, $\sum t(21)=-278.9$, $P_{cluster}=.017$), but no differences between correct and error trials (Figure S6B). This result shows that the link between cerebral response during sleep learning and memory decision cannot be attributed to decisions only based on the identification of image B.

Conversely, we checked whether image A is sufficient to account for EEG differences observed in sleep learning. Trials were defined as correct if the item was correctly identified when it was presented as image A and the item presented as image B was not correctly identified when it was presented as image A. We found an expected modulation of the frontal response for the first and second waves (correct *vs.* baseline: [0.59, 0.87]s, d=0.25, $\sum t(21)=127.5$, $P_{cluster}=.039$ and [1.01, 1.60]s, d=-0.21, $\sum t(21)=-260.9$, $P_{cluster}=.001$; error *vs.* baseline: [0.86, 1.44]s, d=0.18, $\sum t(21)=-210.4$, $P_{cluster}<.001$), and a difference between correct and error trials for the third wave (correct *vs.* error: [3.31, 3.69]s, d=0.28, $\sum t(21)=-102.11$, $P_{cluster}=.031$; Figure S6C). This result confirms that the link between cerebral response during sleep learning and memory decision can be attributed to decisions relying on the identification of image A.

These findings support that sleep learning results mainly from the identification of image A.

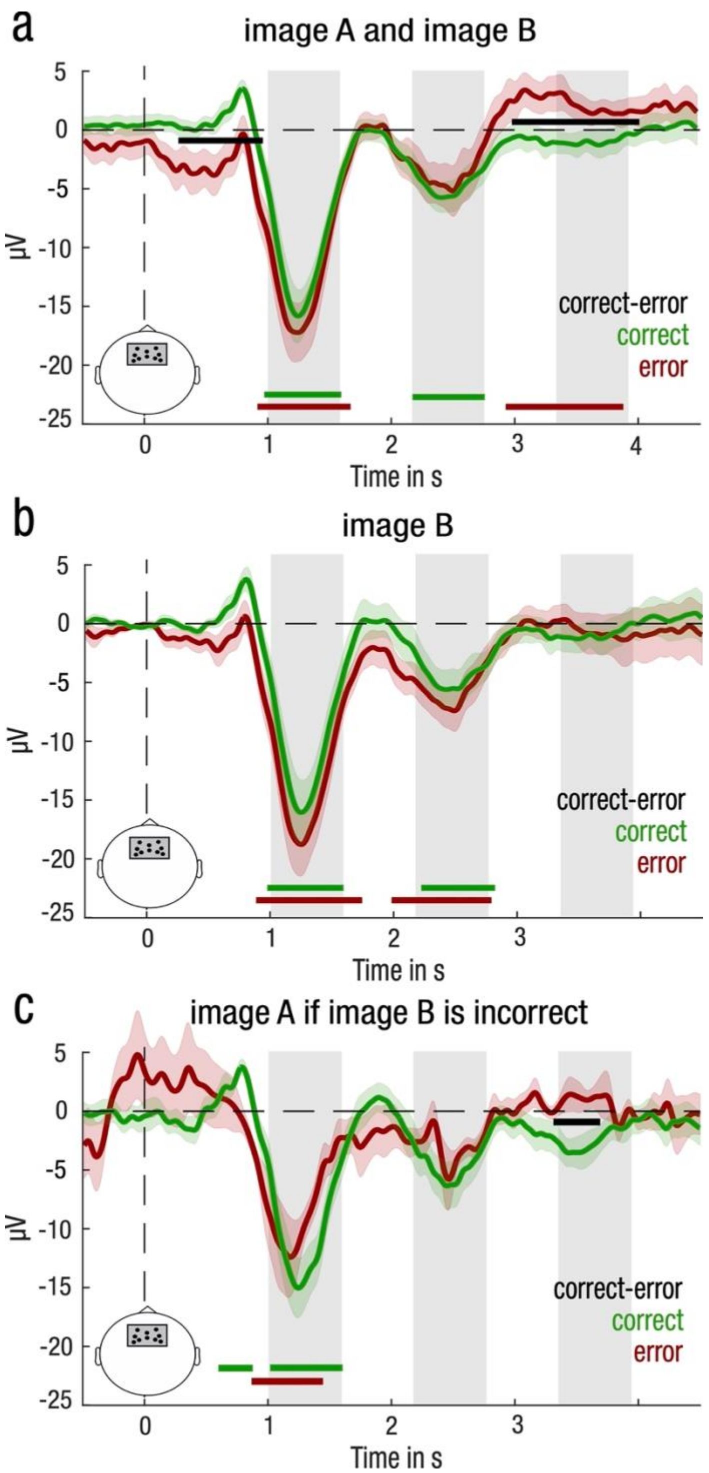

Figure S6. **Differential entrainment of frontal neural responses to stimulation for different definitions of correctly and incorrectly identified items.**

(**A-C**) Mean and standard error of the mean (SEM) are represented respectively with solid lines and shaded areas for the correct (green) and the error (red) trials for correct trials defined as including items from both image A and image B (A), from image B only (B) or image A if image B was incorrectly identified in its own test trial (C). Green, red and black horizontal lines denote significant clusters of neural responses differing from baseline (0, dotted line) for respectively correct trials, error trials, or the difference between both conditions (P<.05 after cluster correction). Grey bars indicate time clusters corresponding to negative half-periods of a slow-wave rhythm at 0.85Hz.

Table S1. **List of the 48 Japanese words used for the experiment**

The English translation of Japanese words is indicated in parenthesis. Please click on the following link to access the auditory and visual versions of each item:

Ame (rain)	Hachi (bee)
Ressha (train)	Uma (horse)
Hikōki (airplane)	Iruka (dolphin)
Jitensha (bike)	Shishi (lion)
Kuruma (car)	Okami (wolf)
Okane (coins)	Nami (wave)
Benjo (toilets)	Hyōzan (ice)
Hōki (broom)	Kaze (wind)
Kane (bell)	Raiu (storm)
Inryou (drink)	Kasai (fire)
Hasami (scissors)	Ringo (apple)
Kage (keys)	Awa (bubble)
Tobira (door)	Denki (electricity)
Sosa (photocopy)	Kokoro (heart)
Shaberu (shovel)	Warai (laugh)
Inu (dog)	Hana (nose)
Ushi (cow)	Meiso (meditation)
Tori (bird)	Seipan (kiss)
Buta (pig)	Tokei (clock)
Fukurō (owl)	Muchi (whip)
Mendori (chicken)	Nezumi (mouse)
Hitsuji (sheep)	Tenshi (angel)
Semi (grasshoper)	Yougan (lava)
Kamo (duck)	Kami (paper)

Table S2. **Summary of stimulation statistics for NREM and REM lists during the sleep learning phase**
The number of repetition of items varied across participants due to difference in sleep patterns and differences in the efficiency of the online sleep scoring across sleep stages. Sleep scoring was performed online (M.K.) and offline by two trained scorers (M.K and D.L.). The scoring efficiency was computed by dividing the number of trials of a given list that were played in the correct sleep phase over the total number of trials that were played during the sleep learning phase. Mean and Standard Error to the Mean (±) are reported.

Number of repetitions	NREM list (16 items)	REM list (16 items)
in NREM sleep (N2 and N3)	40±2.6	1.8±0.38
in REM sleep	0.61±0.21	13±1.2
in Wakefulness (Wakefulness and N1)	0.45±0.12	0.23±0.06
Online scoring efficiency	97±0.5%	85±3.6%

Table S3. **Sleep statistics during the sleep learning phase**
The sleep onset latency was defined as the first apparition of stage NREM2 since the beginning of the sleep phase. Sleep efficiency was calculated as the percentage of time spent in NREM or REM sleep during the sleep phase. Mean and Standard Error to the Mean (±) are reported.

Sleep duration (min)	165±6.5
Duration of light NREM sleep (min)	102±5.7
Duration of deep NREM sleep (min)	23±4.7
Duration of REM sleep (min)	39±3.5
Sleep latency (min)	26±3.9
Sleep efficiency (%)	81±1.8
